# Resistive, Temperature-Independent Metal Oxide Gas Sensor for Detecting the Oxygen Stoichiometry (Air-Fuel Ratio) of Lean Engine Exhaust Gases

**DOI:** 10.3390/s23083914

**Published:** 2023-04-12

**Authors:** Carsten Steiner, Simon Püls, Murat Bektas, Andreas Müller, Gunter Hagen, Ralf Moos

**Affiliations:** Department of Functional Materials, University of Bayreuth, 95440 Bayreuth, Germany

**Keywords:** emissions, gas sensor, engine testing, air-fuel ratio, exhaust gas, barium-iron-tantalate, temperature-independent, oxygen sensitivity, metal oxides, defect chemistry

## Abstract

This study presents a resistive sensor concept based on Barium Iron Tantalate (BFT) to measure the oxygen stoichiometry in exhaust gases of combustion processes. The BFT sensor film was deposited on the substrate by the Powder Aerosol Deposition (PAD) method. In initial laboratory experiments, the sensitivity to *p*_O2_ in the gas phase was analyzed. The results agree with the defect chemical model of BFT materials that suggests the formation of holes $h^{•}$ by filling oxygen vacancies $V_{O}^{••}$ in the lattice at higher oxygen partial pressures *p*_O2_. The sensor signal was found to be sufficiently accurate and to have low time constants with changing oxygen stoichiometry. Further investigations on reproducibility and cross-sensitivities to typical exhaust gas species (CO_2_, H_2_O, CO, NO, …) confirmed a robust sensor signal that was hardly affected by other gas components. The sensor concept was also tested in real engine exhausts for the first time. The experimental data showed that the air-fuel ratio can be monitored by measuring the resistance of the sensor element, including partial and full-load operation modes. Furthermore, no signs of inactivation or aging during the test cycles were observed for the sensor film. Overall, a promising first data set was obtained in engine exhausts and therefore the BFT system is a possible cost-effective alternative concept to existing commercial sensors in the future. Moreover, the integration of other sensitive films for multi-gas sensor purposes might be an attractive field for future studies.

## 1. Introduction

The efficient and sustainable use of fossil fuels is the top priority in terms of conserving finite resources and protecting human health and the environment. For this reason, combustion engines require precise control of the combustion processes in combination with appropriate exhaust gas aftertreatment. One of the most important key parameters is the oxygen stoichiometry or air-fuel ratio *λ*, which has a critical influence on combustion efficiency and also on the performance of catalytic converters for exhaust gas aftertreatment [1,2]. This requires reliable high resolutions gas sensors.

Exhaust sensors in the exhaust pipe are exposed to harsh conditions. High temperatures as well as oxidizing or reducing gases, particles, and long operating times are among the main challenges to ensuring sufficient accuracy and fast response times. At present, sensors based on yttrium-stabilized zirconium dioxide (YSZ) are used almost exclusively in the automotive sector. Such sensing elements use the oxygen ion conductivity of the ceramic, which starts at higher temperatures (>600 °C), to detect the oxygen partial pressure (*p*_O2_). Commercial concepts are based either on a potentiometric (binary Nernst cell) or amperometric sensor effect (wideband lambda sensor) [3,4,5,6].

A disadvantage of potentiometric systems is their limitation to stoichiometric conditions around *λ* = 1, as commonly used in gasoline engines. Operation in lean-burn or diesel engines is therefore not advantageous. The amperometric sensor, on the other hand, covers a larger range of applications. However, the production of the measuring elements is expensive and time-consuming due to the materials used and the complexity of the multi-cell layout for the sensor principle, which is usually necessary since measurements are conducted relative to a reference gas atmosphere (air) [3,4,5,6].

Based on these considerations, another novel sensor concept is presented here. It is therefore based on a planar setup in thick-film technology combined with the powder aerosol deposition method (PAD). The resistive sensor with a sensor film made from the metal oxide Barium Iron Tantalate BaFe_1−*x*_Ta*_x_*O_3–*δ*_ may offer economic advantages for industrial production. In addition, using an appropriate composition (*x* ≈ 0.25), the electrical properties of BFT are independent of temperature [7] and this specific BFT composition is therefore also superior to conventional sensor technologies. Additionally, the higher resistance against sulfurization compared to materials with the same temperature-independent properties like SrTi_1−*x*_Fe*_x_*O_3−*δ*_ may also be a beneficial factor [8]. Moreover, such sensor platforms appear attractive for the integration of additional sensitive layers, allowing also other gas species to be measured simultaneously. The concept is therefore also promising for so-called multi-gas sensors in the field of high-temperature applications.

## 2. Fundamentals

Semiconducting metal oxides change their conductivity at elevated temperatures depending on the *p*_O2_ of the surrounding gas atmosphere. As the sensor material interacts with the oxygen molecules, oxygen is incorporated into or removed from the crystal lattice. Depending on the material and *p*_O2_ in the environment, this interaction is accompanied by a change in the concentration of the conduction electrons or the holes in the material. This interaction, therefore, affects the concentration of majority charge carriers, resulting in a change in the conductivity σ. The physicochemical effect is described by the following equation:

(1) $$\sigma =\sigma_{0}∙e^{\left(-\frac{E_{A}}{k∙T}\right)}∙{\left(p_{O2}\right)}^{m}$$

In this equation, *σ*_0_ is a constant, *E*_A_ is the activation energy of the formation and mobility of the majority charge carriers and *m* represents the material-specific oxygen partial pressure dependency. Furthermore, *k* is the Boltzmann constant and *T* represents the temperature.

Typically, a major issue in the development of metal oxide sensors is their temperature-dependent resistance signal. To overcome this problem, previous studies have focused on material systems with negligible temperature cross-sensitivities due to their particular crystal structure and doping concentrations. Some common examples are Co_1•*x*_Mg*_x_*O [9], SrTi_1−*x*_Mg*_x_*O_3_ [10], La_2_CuO_4_ [11], and SrTi_1−*x*_Fe*_x_*O_3–*δ*_ [12,13,14,15,16]. The latter material has long been considered a promising candidate for the above-mentioned application. However, irreversible inactivation by sulfur oxides during the initial experiments in real exhaust gas remains a challenging problem [12,13,14,17].

Another potential material for temperature-independent sensor applications is Barium Iron Tantalate (BaFe_1−*x*_Ta*_x_*O_3–*δ*_), also known as BFT. Although the material system has been known for some time, there are relatively few publications in the literature [8,18,19,20]. Recent research has been presented by Bektas et al., who have also provided a defect-chemical model and laboratory experiments to apply BFT as a temperature-independent resistive or thermoelectric oxygen sensor material at temperatures above 500 °C. At high *p*_O2_ (>10^−4^ bar), the resistive response of BFT materials is based on the formation of holes $h^{•}$ by filling oxygen vacancies $V_{O}^{••}$ in the lattice [8,16]:

(2) $$\frac{1}{2}O_{2}+V_{O}^{••}↔O_{O}^{x}+{2h}^{•}$$

Following this equation, BFT behaves as a *p*-type semiconductor with a decreasing resistance at higher oxygen concentrations under these conditions. At high *p*_O2_, the concentration of oxygen vacancies is fixed by the concentration of reduced iron cations [Fe′], which act as acceptors. Therefore, the following electroneutrality condition applies [8]:

(3) $$2[V_{O}^{••}]\;\approx \;[{\mathrm{Fe}}^{\prime }]$$

Due to acceptor doping, BFT a dependency on the *p*_O2_*^m^* of *m* = +1/4 is typically observed in experiments under the specified conditions. Across all studies, a stoichiometric tantalum content between 20% < *x* < 30% provides the best temperature-independent properties for sensor applications [7,8,18,21,22]. The temperature-independent behavior of BFT is based on the contrary thermal activation of charge carrier concentration [$h^{•}$] and hole mobility *µ*_h_. While the concentration [$h^{•}$] decreases with higher temperatures (oxidation enthalpy Δ*H*_Ox_ ≈ −0.55 to −0.35 eV), and the mobility of the defect electrons by small polaron hopping increases (migration enthalpies Δ*H*_µ_ ≈ 0.08–0.23 eV. Both effects are observed to balance each other above ≈ 600 °C, resulting in a temperature-independent sensor response with low thermal activation of electrical conductivity (*E*_A_ < 0.1 eV) for BFT materials [8].

Based on these findings, the results presented in this study are based on a composition of BaFe_0.74_Al_0.01_Ta_0.25_O_3–*δ*_ (BFAT25, *x* = 0.25), including 1% alumina to improve thermal stability. From this material, oxygen-sensitive thick films (thickness of approximately 2 to 5 µm) were prepared by the Powder Aerosol Deposition (PAD) method [23]. This study aims to give an overview of the sensor signal, its quality, and reproducibility, and provides the first experiments in real engine exhaust gas.

## 3. Materials and Methods

A total of four independent powder batches of the functional material BaFe_0.74_Al_0.01_Ta_0.25_O_3–*δ*_ were prepared for the investigations, which will be designated as BFAT25-*z* in the following, with *z* being the number of the batch. BFAT25-1 was used for detailed verification of the low-temperature dependency of the resistance within a separate set of measurements. The other three materials (BFAT25-2,3,4) were used for reproducibility studies, further investigations, and the first tests in the real exhaust gas. All batches were produced separately using the mixed-oxide route, similar to [21]. The reactants BaCO_3_ (Alfa Aesar, Haverhill, MA, USA, 99%), Fe_2_O_3_ (Alfa Aesar, 98%), Al_2_O_3_ (Almatis), and Ta_2_O_5_ (Alfa Aesar, 99%) were weighed stoichiometrically. Each batch comprised a total powder quantity of ca. 25 g. Powder mixtures were then homogenized with a planetary ball mill (Fritsch Pulverisette 5, Idar-Oberstein, Germany) with cyclohexane (grinding medium) in a ZrO_2_ milling jar (stabilization: 3.5% MgO) with ZrO_2_ milling balls (Ø 10 mm, stabilization: 5.0% Y_2_O_3_, amount: 230 g). Homogenization was performed in 16 cycles of 15 min each at 180 rpm with a 30 min break between each cycle. The total grinding time was 4 h. After milling, the homogenized powders were dried first in the air for at least one day and then in a furnace at 200 °C for another 24 h. The maximum temperature of the subsequent calcination process of 1350 °C was maintained for 15 h in a chamber furnace in air. The powder preparation was finalized by a 2-step grinding process in the planetary mill. The first step used bigger ZrO_2_ milling balls (Ø 20 mm, stabilization: 5.0% Y_2_O_3_, amount: 5 pcs.) and comprised 5 cycles of 15 min each at 180 rpm and 30 min breaks. The second step again was performed with the smaller ZrO_2_ milling balls (Ø 10 mm, stabilization: 5.0% Y_2_O_3_, amount: 230 g) in 11 cycles, 15 min grinding at 180 rpm, 30 min breaks. Both procedures used again ZrO_2_ milling jars (stabilization: 3.5% MgO) and cyclohexane as a grinding medium. Afterward, the larger agglomerates were eliminated with a 200 µm sieve.

**Figure 1:**
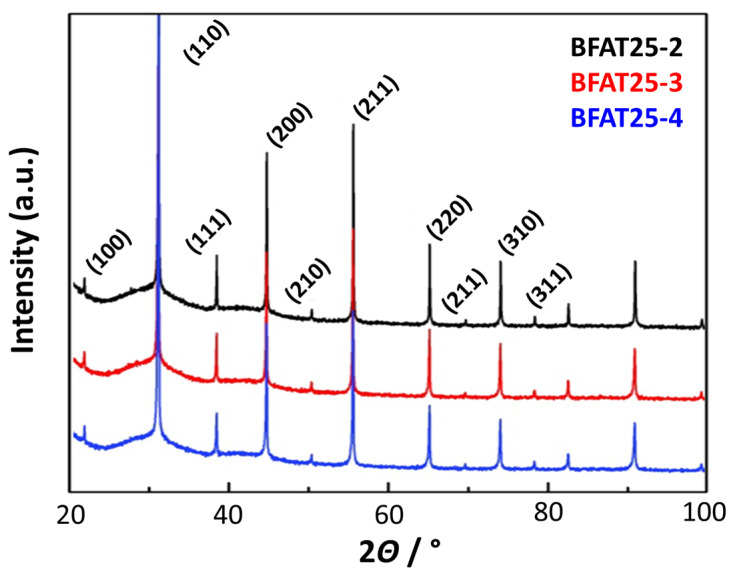
Diffraction patterns of the powder batches BFAT25-2, 3, and 4.

The ceramic powders were then analyzed at room temperature with the *X*-ray diffractometer (XRD, PANalytical X’Pert Pro) with Cu-K_α_ radiation (wavelength: 1.5406 Å) in a 2*Θ*-range from 20° to 100° with a resolution of 0.02°. Figure 1 shows the normalized intensities of the powder batches BFAT25-2,3,4. All materials have a perovskite crystal structure without any occurring secondary phase. In addition, the patterns confirm the identical quality of each powder batch, which also certifies crystal structures and phase purity. A reproducibility study of the electrical properties is therefore reasonable. The data from the BFAT25-1 batch (not shown) provided similar results.

**Figure 2:**
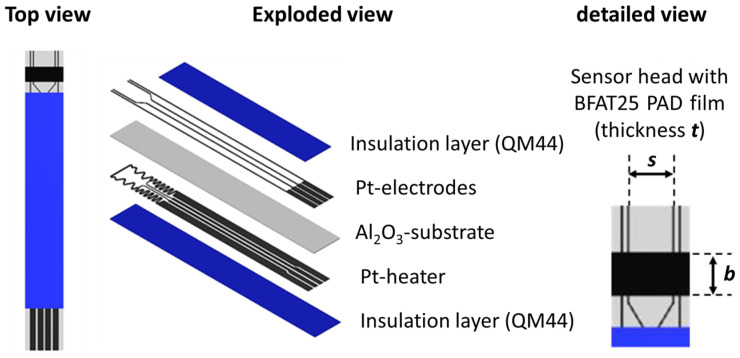
Scheme of the sensor elements with a BFAT25 PAD film.

**Figure 3:**
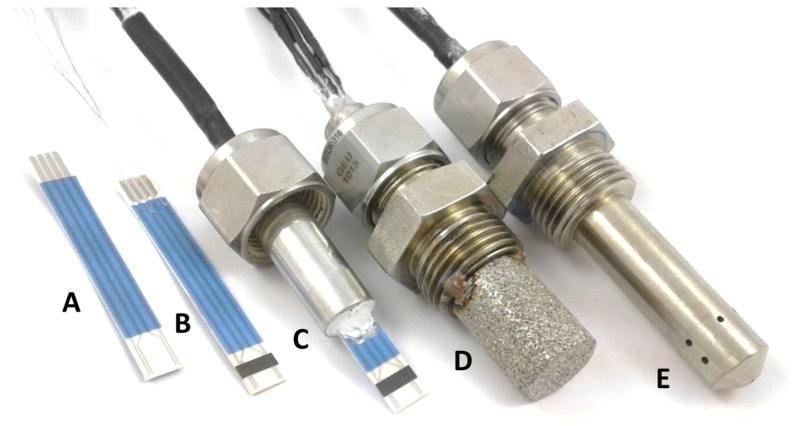
(**A**): Sensor element without BFT film, (**B**): with BFT film, (**C**): mounted in housing without a protection cap, (**D**): sinter metal protection cap added, (**E**): protection cap with small holes instead of a sinter metal cap.

After material analysis, the ceramic powders were processed to sensor elements in several production steps. The structure of such sensing elements is shown schematically in Figure 2 and Figure 3 show the steps from the sensor element to the fully housed sensor device. The sensor element consists of an aluminum oxide substrate (50 mm × 5 mm; thickness: 650 µm) with four platinum electrodes on the front side and a platinum heater structure on the reverse side.

**Figure 4:**
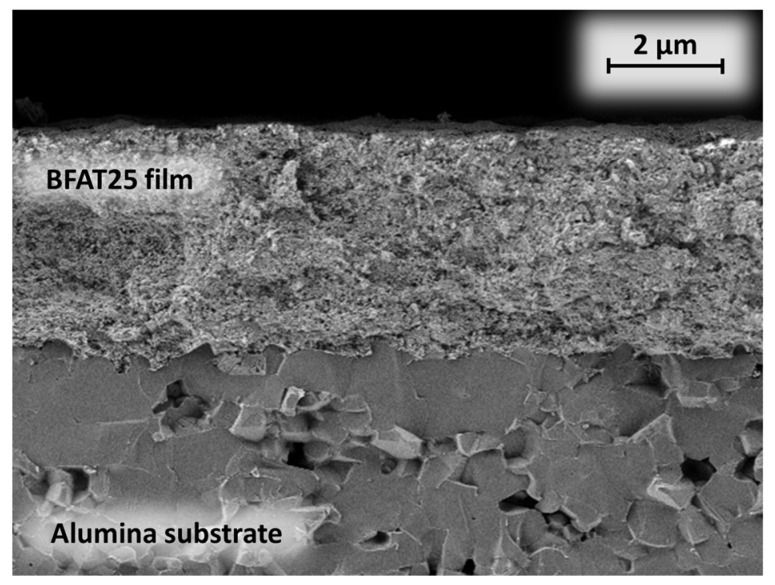
SEM micrograph of the nanocrystalline BFAT25 films on the alumina substrate.

The functional BFT layer was deposited directly from the ceramic powder by using the powder aerosol deposition (PAD) method, which is described in more detail in [23]. The sensing elements were then annealed at 800 °C for 3 h for relaxation as suggested by [24]. The quality and the microstructure of the deposited BFAT25 films were investigated with a scanning electron microscope (SEM, Leo 1530 P V, Zeiss, Oberkochen, Germany). A representative image of the sensor films (sectional view) is shown in Figure 4. In contrast to the coarse-grained alumina substrate, the dense ceramic BFAT25 film has a nanostructured morphology with a typical grain size of some tens of nanometers. The SEM images also confirmed a film thickness *t* of 2 to 5 µm.

Measurements on sensor functionality and reproducibility were conducted in a chamber furnace with a synthetic exhaust gas atmosphere. For further characterization in the laboratory test benches and testing in the real exhaust gas, the sensor elements were contacted with wires and embedded in a stainless steel housing. Including a protective cap, the sensor was then placed directly in the exhaust gas flow. The electrical resistance *R* of the sensor films was recorded in a four-wire technique using a Keithley 2700 digital multimeter with offset compensation. Taking into account the layer geometry, the electrical conductivity can then be determined:

(4) $$\sigma =\frac{1}{R}∙\frac{s}{b∙t}$$

with the distance between the inner electrodes (or “effective” film length) *s*, the film width *b*, and thickness *t*. The geometric dimensions were determined using a perthometer for the thickness *t* and a slide gauge for length *s* and width *b*, respectively. The further geometric dimensions of the sensor films are approx. 3.5 mm (effective length *s*) and 2.4 mm (width *b*). Despite the low grain size, the conductivity in the BFT layer is expected to be unaffected by grain barriers. This assumption is confirmed by a comparison of nanocrystalline PAD layers and sintered samples in [8].

## 4. Results and Discussion

The presentation and discussion of the evaluated data are divided into four parts. First, the results of batch BFAT25-1 are presented. This is followed by the reproducibility study of the three other powder batches BFAT25-2,3,4. Both investigations were carried out in the same measuring system on sensor elements without a protection cap (see Figure 3B,C). The third section comprises the remaining laboratory tests. Here, sensor elements with housing and protection caps were used (Figure 3D,E). Finally, the last section presents the results in real engine exhaust. Both the investigations on batch 1 and the reproducibility studies were carried out in an externally heated gas chamber with an inactive heater of the sensor element. However, during further laboratory measurements and the tests in the exhaust pipe, the sensor was operated self-heated. For this purpose, the platinum heater on the reverse side was operated with an external controller. The required heating characteristic was determined by the prior calibration of each test substrate.

### 4.1. Analysis of Oxygen Sensitivity (BFAT25-1)

For the detailed characterization, a sensor (dimensions: *s* = 3.5 mm; *b* = 2.4 mm; *t* = 3.5 µm) from the BFAT25-1 batch was exposed to different *p*_O2_ at different temperatures (starting with 800 °C, cooling in steps of 25 °C down to 600 °C). Starting with pure nitrogen at each temperature, the oxygen concentration was then increased in intervals of 30 min from 1% to 5%, 10%, 20%, 50%, and 100% O_2_, which leads to oxygen partial pressures of 10^−2^ bar, 5 × 10^−2^ bar, 10^−1^ bar, 2 × 10^−1^ bar, 5 × 10^−1^ bar, and 1 bar, respectively. The gas flow (200 mL/min) contained a constant humidity level of 3%. Between each cycle, the system was flushed with pure nitrogen for two hours.

**Figure 5:**
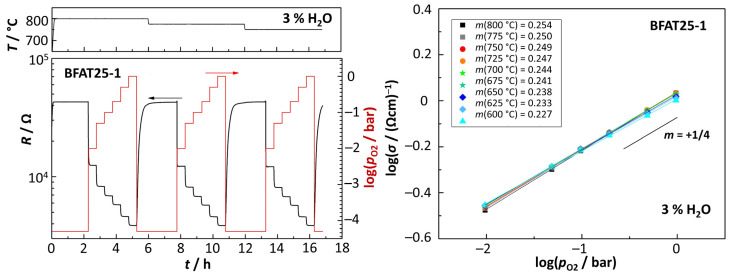
(**left**): electrical resistance of the BFAT25 film at different *p*_O2_ (set points) and temperatures (800 °C, 775 °C and 750 °C); (**right**): calculated *p*_O2_-dependencies *m*.

On the left side, Figure 5 shows the electrical resistance of the BFAT25 film for temperatures 800 °C, 775 °C, and 750 °C. The sensor reaction on the individual *p*_O2_ within each temperature range and on the system flushing with pure nitrogen between the experiments is clearly visible. The sensor element provides a constant and stable signal for each *p*_O2_ and is almost insensitive to changes in temperature. In general, the response time when changing to higher *p*_O2_ is faster than in the opposite direction. The electrical conductivities for each temperature range were calculated from the electrical resistance data and plotted in a doubly logarithmic *σ* vs. *p*_O2_ plot, as is typical for defect chemical analyses [8]. The plot is shown on the right-hand side of Figure 5 together with the calculated slopes *m*.

The measured resistance *R* at a given *p*_O2_ hardly changes with temperature and therefore also the calculated *p*_O2_-dependencies at the different temperatures have similar values. The widely overlapping data confirm the temperature-independent performance of BFAT25 in the temperature range investigated and thus agree with findings in the literature [7,8,18,19,21,22]. The determined slopes of *m* ≈ 1/4 are typical for the acceptor-controlled defect mechanism of Equations (1) and (2). This finding indeed fits the expectations from previous studies that focused on the characterization of the material. Additionally, the orders of magnitude of the measured conductivities are within a reasonable range.

### 4.2. Reproducibility Studies on Oxygen Sensitivity (BFAT25-2,3,4)

As part of the reproducibility studies, two sensors each were prepared from each of the powder batches BFAT25-2,3,4 within one PAD process. Subsequent investigations with a laser scanning microscope provided data about the geometric dimensions of the BFAT films, as listed in Table 1. The electrode distance *s* was obtained from screen-printing, and the width *b* was given by the nozzle movement. It is therefore not astonishing that it is very reproducible. The film thicknesses are in the order of a few micrometers (approx. 2–5 µm). The variations in layer thickness suggest an unsteady deposition process, which varies from sensor element to sensor element.

The electrical characterization of the sensor elements was conducted using the same protocols and equipment as in Section 4.1. Here, two sensors for each powder batch were examined simultaneously in one measurement cycle. To reduce measurement time, the temperature was increased in 50 °C steps. Additionally, the 3% humidity was kept constant. The recorded data show a similar performance of the sensor films throughout the experiments as presented in Figure 5. The measurements, therefore, confirm the statements formulated above regarding signal quality and stability.

**Figure 6:**
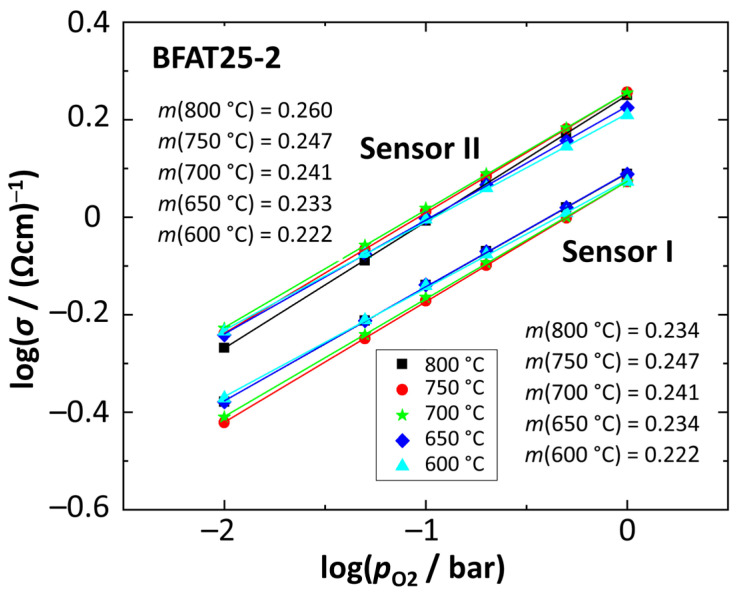
Oxygen sensitivity of the BFAT25 film with the calculated slope *m*.

Based on these investigations, Figure 6 shows the electrical conductivities of two sensors of the BFAT25-2 batch as a function of *p*_O2_, again in a doubly logarithmic representation. The plot also includes the calculated *p*_O2_ dependencies *m*. The difference in conductivity between both sensors is most likely due to uncertainties in determining the thicknesses of the BFAT films. However, the data also from other sensors of this series are within a reasonable range and are also consistent with the values in Section 4.1. To compare the oxygen sensitivities, the calculated slopes *m* are presented in Table 2.

A look at the values shows that sensors from the same batch had identical or nearly identical oxygen sensitivities *m*. Furthermore, there were no statistical outliers within the entire series of measurements. The very small values of the standard deviations indicate that even when comparing different powder batches, there were only small deviations and overall very similar slopes. It is also noticeable that the standard deviation had the highest value at 800 °C and was even smaller at lower operating temperatures. From the data presented, it can be concluded that the material-specific oxygen sensitivity could be reproduced over three batches. The electrical conductivities were also in the same range for all sensors. This is an indicator, that throughout all sensor films, the defect mechanism in Equation (1) is hardly influenced by other factors, such as synthesis parameters, etc., in this experiment. Deviations are due to simplifications in the calculation of conductivity, in particular the assumed film geometry. These results would allow serial production, but a specific one-point calibration of the resistance would be necessary.

### 4.3. Studies on Selectivity of the Sensing Elements

**Figure 7:**
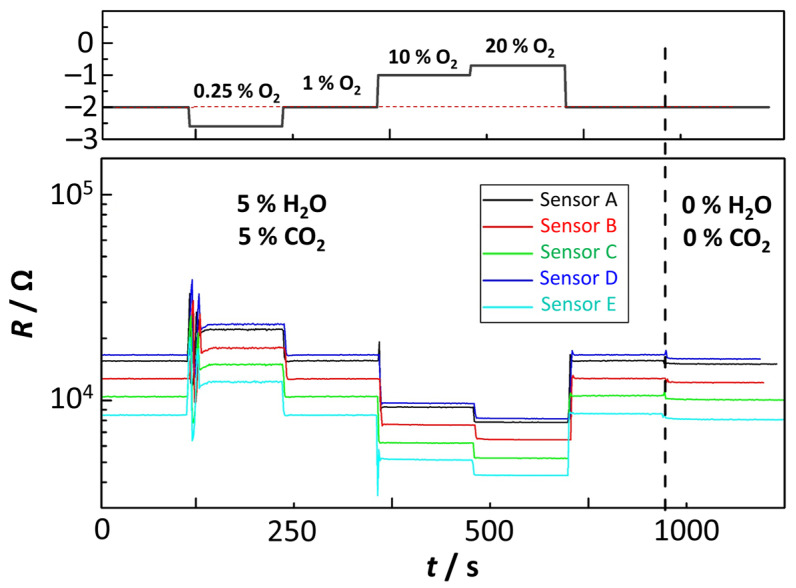
Sensor resistance *R* of several sensors at *T*_Sensor_ = 750 °C and different *p*_O2_.

After the reproducibility studies concerning oxygen sensitivity were completed, the selectivity of the sensing elements to other gas species commonly found in exhausts was investigated. First, the effect of H_2_O and CO_2_ was examined, which is of particular interest because both gases are present in exhaust gases of combustion processes and their concentration is commonly a function of the air-fuel ratio. To provide a more comprehensive view of the cross-effects of other gases on the sensor behavior, this section investigates sensors from all four batches (BFAT25-1,2,3,4). In order to integrate the sensor elements into the intended system and for future investigations in real exhaust gases, the sensor elements were mounted in a housing, but no protection cap was added (Figure 3C). For the experiments presented in this section, the sensors were placed in a heated measuring chamber (180 °C). The operating temperature of the sensor elements (*T*_Sensor_ = 750 °C) was achieved by the integrated Pt-heater structure on the reverse side of the sensor substrate. The gas flow rate was 6 L/min. In the first experiment, a gas atmosphere containing 1% O_2_, 5% CO_2,_ and 5% H_2_O (balanced in N_2_) was fed. During the measurement, the oxygen concentration was changed to 0.25%, 1%, 10%, 20%, and then back to 1%. At the end of each cycle, H_2_O and CO_2_ were turned off, so that the total gas flow consisted only of 1% O_2_ in N_2_. Figure 7 shows the data of the experiments for all sensors. The graph shows the sensor resistance *R* over time for the different gas compositions; the dashed vertical line marks the point in time when the addition of H_2_O and CO_2_ to the gas flow was stopped.

The curves in Figure 7 indicate that the sensors respond as expected to oxygen concentration changes. The sensor response was stable and the time constants to stepwise oxygen concentration changes were low. The addition of 5% H_2_O and 5% CO_2_ to the feed gas did not affect the general response of the sensing elements, except a slightly higher electrical resistance *R* was detected in the presence of these gases. During oxygen concentration variations, no errors or major deviations were observed. Table 3 gives a quantification of the effect of H_2_O and CO_2_ on the sensor resistance. Here, *R*_0_ is the sensor resistance in the presence of 5% H_2_O and CO_2_ and *R* is the resistance without these gases. The presence or absence of the two gases affected the measured sensor resistance by less than 5% for almost all sensing elements in this experiment. Since the change was relatively small, it can be assumed that humidity alone did not have a critical influence on the sensor resistance. The results also agree well with findings from one of our previous studies [21]. Furthermore, it is conceivable that the water concentration itself could influence oxygen sensitivity. However, a critical influence on the oxygen sensitivity seems unlikely considering the low response to the water concentration and the high temperatures. The sensor mechanism of Equation (1) is therefore considered to be the main origin of the sensor effect. For the application as an oxygen sensor in the exhaust gas, it can be further concluded that changes in the air-fuel ratio are not expected to contribute significantly to the sensor signal.

**Figure 8:**
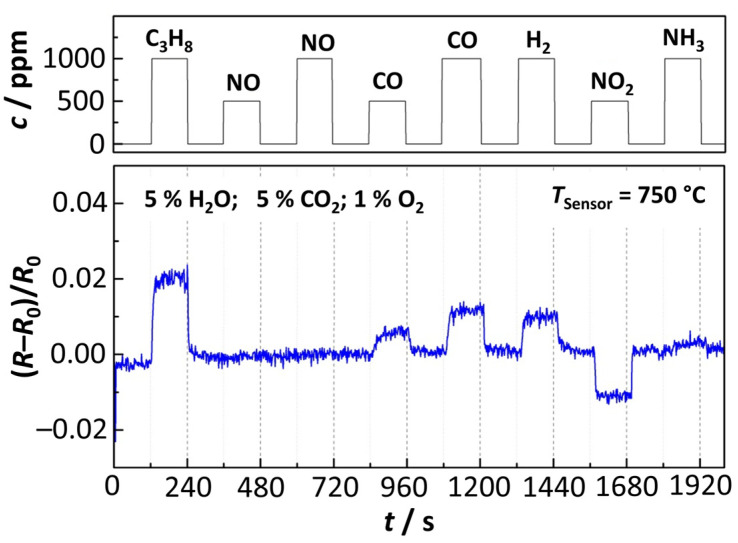
Effect of pollutant concentration on the (relative) sensor resistance of the BFAT film at a sensor temperature of *T*_Sensor_ = 750 °C (sensor B).

Cross-sensitivities to other exhaust components, in particular pollutants, were also investigated. For that purpose, the same setup with a self-heated sensing element (*T*_Sensor_ = 750 °C) was used. The experiment was conducted using Sensor B under a gas composition of 5% H_2_O, 5% CO_2_, 1%, and 10% O_2_ (balance nitrogen). Periodically, concentrations of 500 ppm or 1000 ppm of propane (C_3_H_8_), NO, CO, H_2_, NO_2_, and NH_3_ were added to the feed gas. Each gas component was dosed in intervals of 120 s followed by an equally long section of flushing with the regular H_2_O/CO_2_/O_2_ composition. The experiments were conducted with two different oxygen concentrations of 1% O_2_ and 10% O_2_. At an oxygen concentration of 10%, only a very small change in the resistance *R* was observed that hardly could be resolved from the sensor signal. Higher oxygen concentrations therefore obviously lead to a suppression of cross-sensitivities to many pollutants in the exhaust gas. At an O_2_ concentration of 1%, a quantification of the change in film resistance *R* was possible. Figure 8 shows the relative change in resistance (referred to as *R*_0_ at pure H_2_O/CO_2_/O_2_ composition) when the sensors were exposed to different gas species.

**Figure 9:**
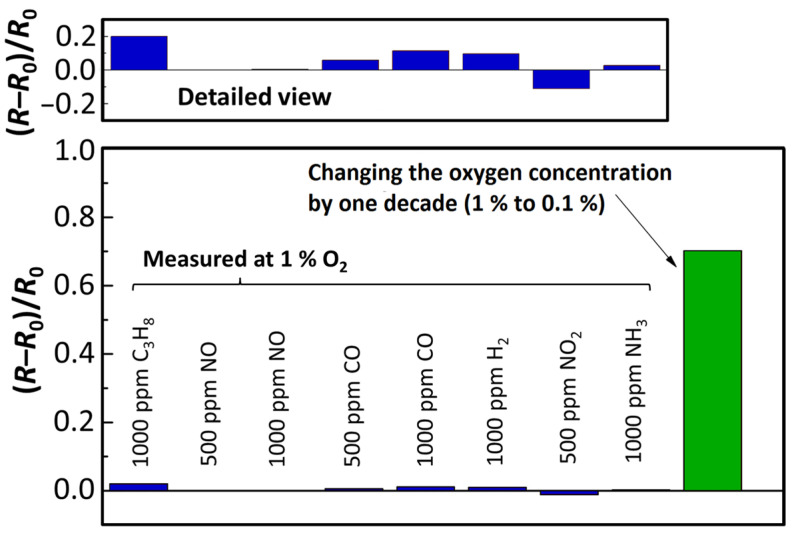
Comparison of cross-sensitivities to different gaseous pollutants measured at an oxygen concentration of 1% (Sensor B data).

The data presented in Figure 8 and Figure 9 compare the relative changes of the resistance of the BFAT film to the different gas species. Additionally, the relative signal amplitude is shown when the *p*_O2_ changes by one decade (from 1% to 0.1%). As the figure demonstrates, the cross-sensitivity of the sensing element to typical components in real exhaust gases was low, even at lower oxygen concentrations. The gases, therefore, do not cause significant deviations in the sensor signal. Furthermore, no irreversible aging or inactivation of the BFAT25 film was observed during operation, which suggests sufficient stability of the sensing material at the applied conditions. The sensor concept based on a BFAT metal oxide film has therefore proved to be suitable in synthetic exhaust gases, with overall high robustness and low cross-sensitivities. Following these results, the approximation of the sensor response characterization by considering the acceptor-controlled (*p*-type) defect behavior seems to be a legitimate and useful method for the first engine experiments.

### 4.4. Tests in Real Exhaust Gas

**Figure 10:**
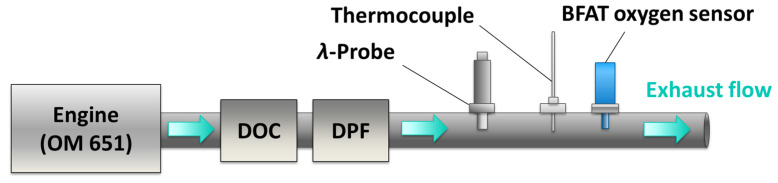
Schematic illustration of the measurement setup on the engine test bench.

After the characterization in the laboratory, initial experiments in real exhaust gas are presented in this section, which were conducted in the exhaust pipe of an engine dynamometer (Mercedes-Benz OM 651, 2.1 l, four-cylinder diesel engine). The setup is shown schematically in Figure 10. Therefore, Sensor C (Figure 3) was placed in the exhaust pipe gas of the engine (Ø 60 mm) and operated at a sensor temperature of *T*_Sensor_ = 750 °C to protect the sensor element from potential condensed water droplets during cold-start and in particular to minimize effects from the dynamic mass flows on the sensor element, a sinter metal protection cap was used as a cover (Figure 3D).

**Figure 11:**
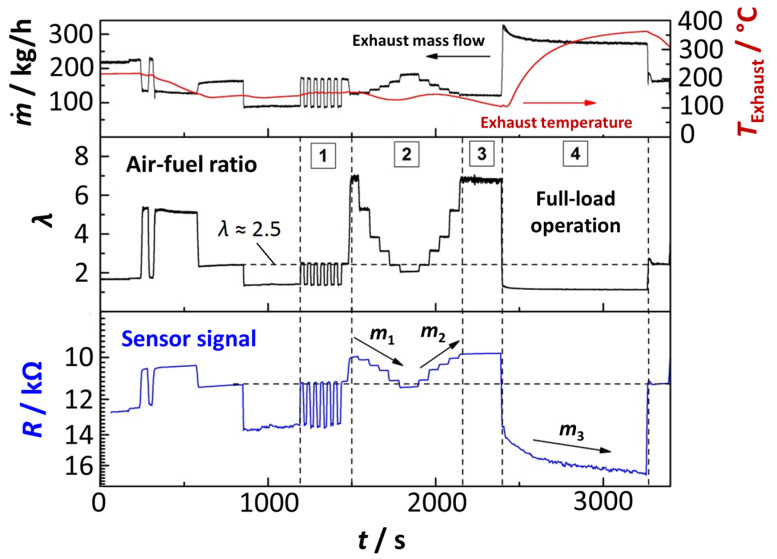
Signals during engine testing: Top: exhaust mass flow $\dot{m}$ and temperature *T*_Exhaust_; Center: air-fuel ratio *λ*; Bottom: Sensor resistance *R*.

The sensor was located downstream of a diesel oxidation catalyst (DOC) and a diesel particulate filter (DPF). A thermocouple and a commercial UEGO sensor (YSZ wide band oxygen sensor acc. to [3]) were placed next to the sensor position to measure the exhaust temperature *T*_Exhaust_ and the air-fuel ratio *λ*. The exhaust mass flow $\dot{m}$ was also recorded. The experimental procedure was started after a warm-up period of several minutes. During testing, the engine load and speed (rotations per minute, rpm) were varied at several intervals, creating a field of different exhaust mass flows, temperatures, and oxygen stoichiometries. Figure 11 presents the data from the engine tests over time. The top plot includes the measured temperature and the exhaust mass flow. The recorded *λ*-signal is presented in the center of the figure. Finally, the resistance signal from the metal oxide sensor can be found in the bottom plot. For the latter, an inverse, logarithmic representation was used for better comparability to the *λ*-signal because this assignment reflects the correlation between conductivity *σ* and the *p*_O2_ (see Equation (1)).

Figure 11 shows clearly that the resistive sensor signal is in line with the lambda sensor in most operating conditions. As in the laboratory measurements, also no irreversible inactivation of the sensor was detected in the real exhaust gas during the engine tests. Furthermore, the response of the resistive sensor has a low time constant and quickly follows changing oxygen stoichiometries. The metal oxide sensor is also reliable during alternating changes between two operating states (Figure 11, Section 1) and provides a sufficient resolution. In particular, at higher lambda values (Figure 11, Section 3) the resistive sensor shows less noise and therefore better signal quality.

During the experiment, various operating conditions with step-wise changing air-fuel ratio *λ* values were also performed (Figure 11, Section 2) as well as a full-load section (Figure 11, Section 4). From the data collected, the oxygen sensitivity *m* was calculated for the descending or ascending stages and at full load. These oxygen sensitivities for the individual segments of the experiment will be referred to as *m*_1_, *m*_2_, and *m*_3_ in the following and are physically identical to the subordinate oxygen sensitivity *m* (Equation (1)). The *p*_O2_ for the evaluation was derived from the UEGO sensor signal. An oxygen sensitivity of *m*_1_ = 0.250 was determined for the descending air-fuel ratio and a slope of *m*_2_ = 0.272 for the ascending *λ*. During the full-load section, an oxygen sensitivity was observed to be *m*_3_ = 0.248. At first glance, the resistance value seems to drift to lower values. However, one can hardly see the small *λ* decrease, which results in a huge *p*_O2_-change, due to the non-linear *λ*(*p*_O2_) behavior [25,26]. The three determined oxygen sensitivities are similar and agree well with former laboratory investigations (Section 4.1 and Section 4.2), even if the experimental setup and the sensor operation mode were different in both investigations. While in the laboratory experiment, the sensor element was kept at a homogenous temperature in a chamber furnace, and the sensor element was self-heated (*T*_Sensor_ = 750 °C) during engine testing using the planar heater on the reverse side. For the measurements in the engine exhaust, changes in the sensor temperature distribution are therefore expected due to variations in the flow and temperature conditions in the exhaust gas. The experimental data in Figure 11 show that $\dot{m}$ and *T*_Exhaust_ indeed varied during the individual segments for *m*_1_, *m*_2_, and *m*_3_. However, considering the temperature-independent nature of the BFAT conductivity (Figure 5), oxygen sensitivities *m* similar to those in Figure 5 can still be expected and are indeed found in the experiment. These findings, even more, confirm that the sensor response is independent from the temperature and the sensor response in the exhaust gas can be described sufficiently by the *p*-type defect chemistry of BFAT. In return, this confirms the above assumption that humidity does not significantly contribute to the sensor signal.

During the whole experiment, an operating mode with a lambda value of *λ* ≈ 2.5 was applied several times and was also set at the end of the test procedure (indicated by the horizontal line in Figure 11). As the data show here, the resistive sensor returns to the same resistance values whenever this operation point is set throughout the experiment. Even if these data can only give a first impression, they give evidence of a certain reproducibility and stability of the sensor. In summary, the first real exhaust gas test of the resistive oxygen sensor with a BFAT25 film produced by the PAD method was successful. The observed oxygen sensitivities confirm the laboratory experiments and suggest that the presented sensor concept is suitable for application in typical diesel engine exhausts. It can be expected that an application in exhausts from biomass combustion is also possible.

## 5. Conclusions

In this study, a novel resistive sensor concept to measure the oxygen concentration of exhaust gases of combustion processes was presented. The sensor is based on BaFe_0.74_Al_0.01_Ta_0.25_O_3−δ_ films deposited by the powder aerosol deposition (PAD) method. Detailed experiments between 600 °C and 900 °C confirmed the temperature independency and the oxygen sensitivity know from previous studies [7,8,18,21,22]. A first analysis of three other powder batches found stable and reproducible results for all sensor elements in laboratory conditions. The experiments suggested a high degree of consistency (reproducibility), in particular concerning the oxygen sensitivity and the conductivity values of the samples. Deviations here are most likely due to the erroneous estimation of the thickness of the sensitive film, which could be refined in future investigations. In addition, investigations were carried out on the cross-sensitivity of the sensing elements to major pollutants of combustion exhaust gases. The results showed only minor cross-sensitivities compared to the desired response to oxygen in the exhaust gas. In a final experiment, the sensor concept was tested on an engine dynamometer under real exhaust gas conditions (typical diesel exhaust pipe application). With the metal oxide sensor, the oxygen concentration could successfully be monitored during step-wise changing engine operation modes. In general, a chemical and physical stable sensor signal was observed without any signs of aging or inactivation throughout all laboratory and engine experiments. The positive results suggest that the present sensor concept is promising and suitable for measurements in exhaust gases. Nevertheless, further research in a wider operation field and under more dynamic conditions together with extended monitoring of the sensor lifetime and analysis of potential aging mechanisms is recommended to prove that the sensor concept is competitive with today’s YSZ-based sensors. Overall, the sensor concept shows promising results and could also be a cost-effective alternative to existing commercial sensors. Furthermore, the integration of other sensitive films into the sensor concept is simple, which makes the sensor also interesting in the development of so-called multi-gas sensors to simultaneously determine the concentrations of different gases.

## Figures and Tables

**Table 1:** Geometric dimensions of the BFAT25 films.

Batch	Sensor-#	Thickness *t*/µm	Width *b*/µm	Distance *s*/µm
BFAT25-2	I	3.1	2450	3508
	II	2.3	2443	3499
BFAT25-3	I	4.0	2445	3513
	II	2.6	2447	3507
BFAT25-4	I	4.4	2432	3534
	II	5.1	2443	3511
Mean		3.6	2450	3512
Std.-Deviation		1.0	14.22	10.73

**Table 2:** Oxygen sensitivities *m* of the BFAT25 films.

Batch	Sensor-#	*m* (800 °C)	*m* (750 °C)	*m* (700 °C)	*m* (650 °C)	*m* (600 °C)
BFAT25-2	I	0.234	0.247	0.241	0.234	0.222
	II	0.260	0.247	0.241	0.233	0.222
BFAT25-3	I	0.260	0.246	0.239	0.231	0.218
	II	0.259	0.246	0.240	0.232	0.218
BFAT25-4	I	0.262	0.249	0.244	0.236	0.225
	II	0.262	0.249	0.243	0.235	0.224
Mean		0.261	0.248	0.242	0.234	0.222
Std.-Deviation		0.0244	0.0013	0.0017	0.0017	0.0027

**Table 3:** Effect of H_2_O and CO_2_ on the electrical resistance *R* of the BFAT25 film.

Signal	Sensor A (BFAT25-1)	Sensor B (BFAT25-2)	Sensor C (BFAT25-2)	Sensor D (BFAT25-3)	Sensor E (BFAT25-4)
***R*/kΩ**	15.041	12.222	10.121	15.954	8.105
***R*_0_ (H_2_O and CO_2_)/kΩ**	15.608	12.753	10.555	16.685	8.604
***R*/*R*_0_**	0.964	0.958	0.959	0.956	0.942

## Data Availability

All relevant data presented in the article are stored according to institutional requirements and as such are not available online. However, all data used in this paper can be made available upon request to the authors.
